# Vertical Dentofacial Skeletal Divergency Is Not Linked with Oral Health-Related Quality of Life

**DOI:** 10.3390/jcm13030665

**Published:** 2024-01-24

**Authors:** Dinis Pereira, Vanessa Machado, João Botelho, Carolina Lemos, José João Mendes, Ana Sintra Delgado

**Affiliations:** 1 Orthodontics Department, Clinical Research Unit (CRU), Egas Moniz Center for Interdisciplinary Research (CiiEM), Egas Moniz School of Health and Science, 2829-511 Caparica, Portugal; adelgado@egasmoniz.edu.pt; 2 Clinical Research Unit (CRU), CiiEM, Egas Moniz School of Health and Science, 2829-511 Caparica, Portugal; vmachado@egasmoniz.edu.pt (V.M.); jbotelho@egasmoniz.edu.pt (J.B.); jmendes@egasmoniz.edu.pt (J.J.M.); 3 Population Studies Department, Institute of Biomedical Sciences Abel Salazar (ICBAS), UniGENe, Institute for Molecular and Cell Biology (IBMC), Instituto de Investigação e Inovação em Saúde (i3S), University of Porto, 4050-313 Porto, Portugal; clclemos@ibmc.up.pt; 4 UniGENe, Institute for Molecular and Cell Biology (IBMC), University of Porto, 4050-313 Porto, Portugal; 5 Instituto de Investigação e Inovação em Saúde (i3S), University of Porto, 4050-313 Porto, Portugal

**Keywords:** oral health-related quality of life, dentofacial skeletal divergency, malocclusion, orthodontic treatment

## Abstract

The aim of this study is to assess how vertical skeletal malocclusion affects oral health-related quality of life (OHRQoL) among a sample of individuals comprising adolescents, young adults, and adults seeking orthodontic treatment. From January 2019 to March 2020, participants were consecutively enrolled. The assessment of OHRQoL involved measurement using the oral health impact profile (OHIP-14). Lateral cephalograms were performed to measure the vertical skeletal divergency with four cephalometric measurements. Descriptive and inferential statistical analyses were performed. The Mann–Whitney test was applied to compare OHRQoL scores according to the vertical dimension category. The mean age of the participants ranged between 30.3 ± 14.9 and 29.9 ± 14.4 and there was a majority of female participants, between 64.1% and 65.9%. There were no statistically significant differences observed between hyperdivergent and normodivergent groups in either the total score or any domain of the OHIP-14 questionnaire. Individuals with hyperdivergent facial morphology did not show a reduced OHRQoL compared with a normodivergent facial type.

## 1. Introduction

Quality-of-life study has become a rapidly expanding research field in medical and dental research [1,2]. Comprehensively, quality of life is a wide and complex concept, encompassing the influence of individuals’ physical health, psychological state, social relationships, and environmental factors [3]. Consequently, numerous questionnaires have been created and validated to assess the impact of oral conditions and treatments on oral health-related quality of life (OHRQoL) [4]. Notwithstanding, the oral health impact profile (OHIP) created in 1994 is internationally and widely used to ascertain OHRQoL. It was first developed with 49 questions (OHIP-49) [5], and was, however, later shortened to a version with 14 items (OHIP-14) for convenient epidemiological application [6]. OHIP-14 is a well-validated questionnaire and is considered reliable [7,8]. 

Dentofacial deformities such as mandibular prognathism, bimaxillary prognathism or retrognathism, and maxillary vertical excess, are frequently the result of genetic conditions that influence cranio-facial development and can be treated involving orthodontics and orthognathic surgery in adults [9,10]. Patients affected by severe skeletal malocclusions or dentofacial deformities often report a wide range of oral health impacts that significantly influence their OHRQoL [11,12,13]. In this context, dentofacial deformities may lead to patients’ lower self-esteem, social handicap, and psychological distress and cause dissatisfaction with their facial appearance [14,15,16]. Importantly, it is not always clear to distinguish which skeletal malocclusions cause more OHRQoL impairment [17].

Hyperdivergent facial types show significant esthetic involvement with specific characteristics, such as anterior open bite, gummy smile, and excessive lower facial height [18,19]. In addition, studies have reported that these individuals have less facial attractiveness [20,21]. Evidence shows that facial attractiveness is highly valued in modern society and has a significant influence on social behavior, such as in picking a partner [22,23]. Interestingly, research is still scarce to ascertain the cause–effect of the possible association between OHRQoL and skeletal vertical divergence. 

Hence, we aimed to investigate the impact of self-reported OHRQoL, between individuals with hyperdivergent and normodivergent vertical skeletal patterns, using four vertical cephalometric measurements, in a sample of patients seeking orthodontic treatment.

## 2. Materials and Methods

The Egas Moniz Ethics Committee granted approval for this project (Approval no. 769) and all procedures were conducted in strict accordance with guidelines outlined in the Helsinki Declaration of 1975, as revised in 2013. This investigation was carried out following the Strengthening the Reporting of Observational Studies in Epidemiology (STROBE) guidelines (Table S1) [24].

### 2.1. Study Design

This secondary analysis involved a consecutive sample of patients seeking orthodontic treatment in Egas Moniz University Clinic (EMUC), from January 2019 to March 2020. Regarding the primary study, starting with an initial cohort of 405 patients who had prearranged orthodontic consultation, 93 were deemed eligible for this sample [25]. Every participant in this study or their respective legal guardian willingly provided their signed informed consent. To ensure confidentiality, all data collected were recorded in a dedicated database designed exclusively for this research, with each participant assigned a unique coded number.

### 2.2. Participants and Eligibility Criteria

In accordance with the recommendation suggesting OHIP-14 for patients aged 15 years and older [26], the criteria for exclusion were as follows: individuals under the age of 15; participants who had undergone or were undergoing orthodontic treatment; patients with any developmental or craniofacial abnormalities; presence of a deep periodontal pocket (periodontal pocket depth ≥ 4 mm); untreated dental caries; inability to engage in the survey. The collection of data involved a combination of face-to-face interviews and clinical examinations.

### 2.3. Sociodemographic and Clinical Orthodontic Questionnaire

All participants included had the following complete records: sociodemographic status according to a self-reported questionnaire, including age and gender, and a cephalometric radiograph obtained using the digital Orthophos XG 5 DS/Ceph (Sirona Dental System, Long Island City, NY, USA) at the Radiology Department at the EMUC. 

The assessment of OHRQoL was conducted using the OHIP-14 Portuguese version, which was validated and had good precision [27]. The OHIP-14 survey encompasses seven domains that evaluate oral health impact: psychological discomfort, physical disability, functional limitation, physical pain, psychological disability, handicap, and social disability. Within each domain, two questions were applied and participants provided ratings on a 5-point Likert scale with the following codes: 0—never; 1—hardly ever; 2—occasionally; 3—fairly often; and 4—very often. The total score for the OHIP-14 questionnaire was calculated by summing the scores from the 14 questions, ranging from 0 to 56. A higher total score indicates a greater extent of negative impacts and a lower level of OHRQoL [6].

### 2.4. Measurements on Cephalograms

**Figure 1:**
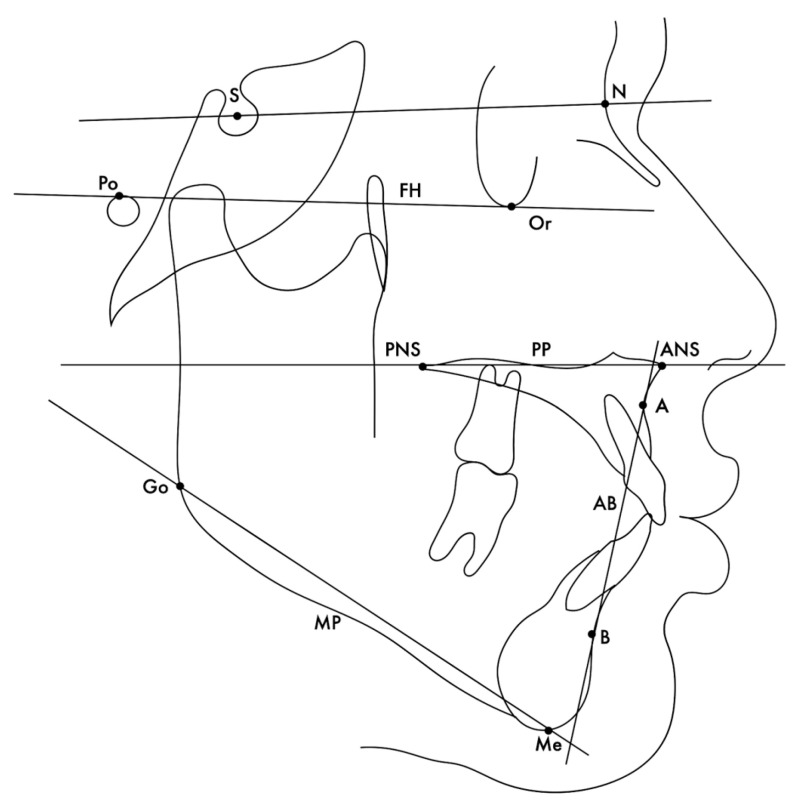
Cephalometric tracing. Lines are constructed to join the landmarks for analysis of angular relationships.

All reference points or anatomical structures were measured by a single operator (D.P.). Cephalometric software (OrisCephMac Ver. 8.3, Milan, Italy) was used for the cephalometric evaluation, and the magnification factor was standardized a priori as 0%. The vertical skeletal pattern was defined according to four vertical angular classifications: (1) sella–nasion plane–mandibular plane (SN-MP); (2) Frankfort horizontal plane–mandibular plane (FH-MP); (3) palatal plane–mandibular plane (PP-MP); (4) overbite depth indicator (ODI), as the sum of the angles formed by the AB plane–mandibular plane and Frankfort horizontal plane–palatal plane (Figure 1). The normodivergent skeletal pattern was defined with a SN-MP angle between 27.0° and 37.0° [28], FH-MP angle between 21.6° and 30.2° [29], PP-MP angle between 19.0° and 31.0° [30], and an ODI angle between 68.4° and 80.6° [31]. The hyperdivergent skeletal pattern was defined with an SN-MP angle ≥ 37.1° [28], FH-MP angle ≥ 30.2° [29], PP-MP angle ≥ 31.1° [30], and ODI plane ≤ 68.3° [31].

### 2.5. Measurement Reliability and Reproducibility

In order to ensure the reproducibility of measurements, one examiner (D.P.) underwent training and calibration with another examiner (V.M.) as the gold standard. Ten randomly selected cephalometric radiographs, obtained from individuals at the Department of Orthodontics who were not part of the study sample, were assessed. Both examiners performed the cephalometric analysis to assess the reproducibility between examiners. After a two-week interval, the same set of ten cephalometric radiographs were reanalyzed by the examiner (D.P.) to evaluate reproducibility within the same examiner. The inter-examiner and intra-examiner correlation coefficients both demonstrated very good results, with kappa scores of 0.94, indicating excellent concordance.

### 2.6. Statistical Analysis

Descriptive and inferential statistical methodologies were applied. Missing data were not identified in the present study, therefore missing data management was not required. The assessment of quality of life using OHIP-14 was calculated as a continuous measure and correspondent descriptive measures (mean and standard deviation [SD]) were computed. We confirmed the lack of data normality; therefore, we applied nonparametric statistical tests. The Mann-Whitney test was applied to compare OHRQoL scores according to the vertical dimension category. To compare hypothetical sagittal differences according to the vertical dimension category, we compared the mean value of the A point–nasion–B point angle. Then, adjusted logistic regression was used to explore the impact of vertical dimension category (normodivergent vs. hyperdivergent) on the total and each domain of the OHIP-14. These models were adjusted for sex and age according to previous differences observed in the primary analysis of the data, as already mentioned and published. For each model, we reported standard error (SE) and the *p*-value. Data were analyzed using R (version 4.0), and we set a level of significance of 5%.

## 3. Results

**Figure 2:**
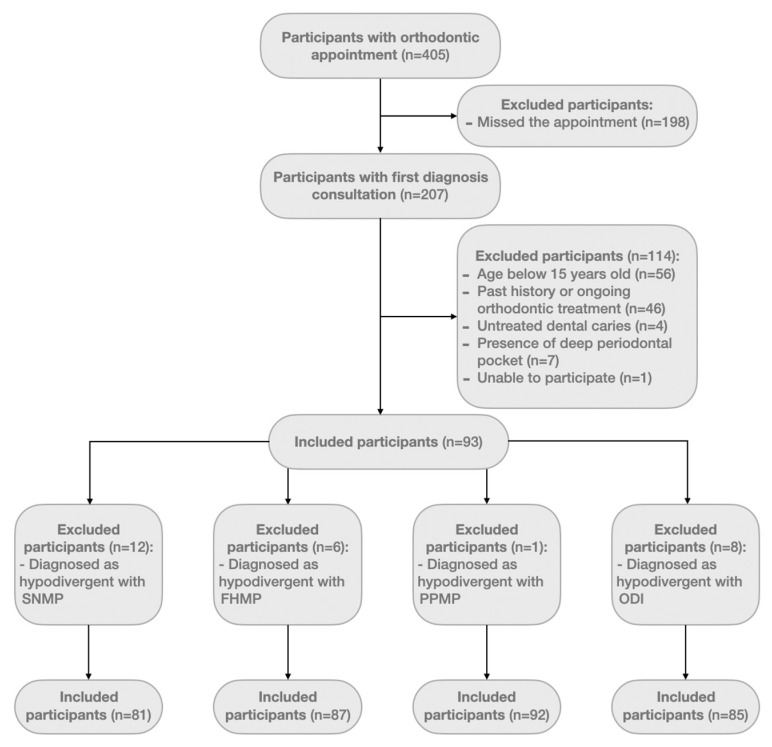
Flow of participants.

Out of the initial sample, a cohort of 93 participants (33 males and 60 females) were included in the study, aged 15 to 60. From these 93, after assessing each cephalogram, patients classified as hypodivergent were excluded, considering each cephalometric angle. Final criteria for inclusion were specifically determined according to each cephalometric measure (Figure 2).

Considering the four cephalometric measures, the mean age of the participants ranged between 30.3 ± 14.9 and 29.9 ± 14.4 and there was a majority of female participants (between 64.1% and 65.9%) (Table 1).

The two groups differed primarily in hyperdivergency, but not in sagittal characteristics. The A point–nasion–B point angle differed but not significantly among the classifications. The ODI was the only classification where hyperdivergent people had a lower sagittal angle (2.0° ± 2.8) than normodivergent (4.2° ± 2.3; *p* = 0.143), while for the remaining categories, hyperdivergent tended to present higher sagittal angles (Table S2).

Comparing the total OHIP-14 scores between the four cephalometric measures, SN-MP and FH-MP hyperdivergent groups had higher scores. Conversely, PP-MP and ODI normodivergent groups had higher scores. There were no significant differences observed between hyperdivergent and normodivergent groups in the correlations for either the OHIP-14 total score or any of the seven domains (Table 2, Table 3, Table 4 and Table 5).

Furthermore, we analyzed the association of age and sex, comparing OHIP-14 scores between hyperdivergent and normodivergent groups (Table 2, Table 3, Table 4 and Table 5). Notably, only the comparison of SN-MP between hyperdivergent and normodivergent groups showed significant impact on OHRQoL, specifically, on OHIP-14 total score (*p* = 0.010) and psychological discomfort (*p* = 0.010) (Table 2).

## 4. Discussion

In the studied population, no significant impact of vertical skeletal dimension was observed on OHRQoL in patients seeking orthodontic treatment. To the best of our knowledge, only one previous study has investigated this association [32]. These findings are relevant because they extend the vertical dimension classification analysis, increasing the robustness of a lack of association. We further shed light on the nonexistent correlation between one of the main malocclusion parameters and OHRQoL.

One study reported that hyperdivergency may have a small but significant overall effect on OHRQoL in orthodontic patients. Moreover, the most important difference in the OHIP-14 scale between individuals with hyperdivergent and normodivergent facial types was in the social disability domain. Nevertheless, this study defined hyperdivergent only by an SN-MP angle greater than two standard deviations from the norm, or in other words, higher than 42°. Additionally, the sample included patients before, during, and after orthodontic treatment [32]. In our opinion, the findings above could be explained due to the inclusion criteria for the hyperdivergent group including only patients with severe vertical discrepancy. Furthermore, evaluating patients during or after orthodontic treatment could influence patient perception, since they might have more self-consciousness about their facial and skeletal condition.

Previous studies found that patients with severe dentofacial deformities experience OHRQoL improvement after orthognathic surgery treatment [33,34,35]. Importantly, one study investigated how different types of deformities influenced the domains assessed by the OHIP-14 questionnaire and reported that patients with class I dentofacial deformities who underwent orthognathic surgery for the correction of vertical or transversal (asymmetry) discrepancies obtained significant improvements in the psychological disability domain, while no significant changes were observed in the other domains [3]. These results underline the fact that vertical skeletal divergence had an impact on only one domain of OHIP-14. Furthermore, it is worth mentioning that only patients with severe skeletal discrepancies met the criteria for orthognathic surgery treatment. 

Comprehensively, it is evident that dentofacial deformities can greatly influence an individual’s life. This impact is not solely determined by the physical aspect of the deformity but also by the individual’s past experiences, psychological constitution, and personality traits. Therefore, the magnitude of the impact may not necessarily correspond directly to the severity of the deformity [36].

Conversely, another relevant variable could be the impact of other malocclusion characteristics on an individual´s OHRQoL. Skeletal hyperdivergent individuals or those with long faces usually have specific dental and facial esthetic concerns, including gummy smile, greater lower facial height, class II malocclusion, posterior crossbite, and anterior open bite [18,19,37]. Previous studies found an association between anterior open bite and impairment of OHRQoL [17,38]. In this context, future research should address the malocclusion variability of hyperdivergent facial type on OHRQoL.

Evidence shows a relationship was found between Class II and Class III malocclusion and the vertical skeletal pattern. There is a tendency toward skeletal compensation with both vertical and sagittal malocclusions [39]. Nevertheless, sagittal skeletal malocclusion was not a confounding variable in our study. In addition, another study also reported that there was no sagittal influence when comparing the impact on OHRQoL between hyperdivergent and normodivergent facial patterns [32].

Concerning the precise definition of facial attractiveness, it is debatable, with opinions differing substantially between clinicians and lay people [40]. Studies found that patients with hyperdivergent facial types reported lower facial attractiveness [20,21,41]. Specifically, one study showed that satisfaction with facial appearance is higher for the general public than for pretreatment orthodontic patients. Orthognathic patients showed the lowest satisfaction levels [42]. Notwithstanding, one study reported that laypeople and orthodontists considered that two-thirds of the long-face sample have an acceptable, pleasant, or very pleasant facial appearance. Interestingly, this study also showed that for lay people, the absence of lip seal (36.2%), followed by exposure of the incisors (28.07%) and eyes (12.31%), were the characteristics most cited for lower facial attractiveness. The absence of lip seal is usually a characteristic in hyperdivergent patients due to an increase of the lower third of the face [43]. Thus, cephalometric measurement of lower anterior facial height with the PP-MP angle should be considered, in order to better evaluate the impact of skeletal hyperdivergency on OHRQoL [44]. 

On the other hand, dentofacial deformity has been considered the most difficult oral condition to measure [45], likewise because it involves a subjective assessment of what constitutes normal esthetics [46]. There are several skeletal cephalometric parameters to assess the vertical skeletal pattern of an individual. Consequently, we used four cephalometric measurements of vertical skeletal divergency in our study. However, various measurements showed different results and a reliable diagnosis is hard to define. Nevertheless, one study compared the accuracy of various cephalometric vertical skeletal measurements and reported that SN-MP and FH-MP had the highest validity. PP-MP angle showed a moderate correlation with the other skeletal angles [44]. Therefore, more studies should investigate the performance of various cephalometric parameters for the assessment of vertical skeletal patterns. 

### Strengths and Limitations

Our study has certain limitations. The use of cross-sectional study design limits our ability to establish inferences about cause-and-effect relationships. However, this study focuses on exploring the complex association among various factors contributing to OHRQoL. The sample size was a limitation, warranting caution in interpreting the results due to the limited validity. Future studies with larger sample sizes are needed to confirm these findings. In spite of that, it is worth noting that this study involved a 15-month inclusion period, followed rigorous and up-to-date guidelines, and had a consecutive design, adding some value to our findings. Additionally, we employed well-established tools for measuring OHRQoL (OHIP-14) and applied four cephalometric measurements widely described in the literature.

## 5. Conclusions

Comparing hyperdivergent and normodivergent facial patterns in this study population, vertical skeletal divergency malocclusion does not have an impact on OHRQoL. Notwithstanding, future clear criteria on the definition of skeletal vertical divergency are warranted to ascertain the impact on OHRQoL.

## Figures and Tables

**Table 1:** Gender and age range according to hyperdivergent or normodivergent pattern, for overall participants.

Variable	SN-MP		Total (*n* = 81)	FH-MP		Total (*n* = 87)	PP-MP		Total (*n* = 92)	ODI		Total (*n* = 85)
	Hyper (*n* = 27)	Normo (*n* = 54)		Hyper (*n* = 45)	Normo (*n* = 42)		Hyper (*n* = 39)	Normo (*n* = 53)		Hyper (*n* = 28)	Normo (*n* = 57)	
Female, *n* (%)	21 (77.8)	31 (57.4)	52 (64.2)	27 (60.0)	30 (71.4)	57 (65.5)	25 (64.1)	34 (64.2)	59 (64.1)	15 (53.6)	41 (71.9)	56 (65.9)
Male, *n* (%)	6 (22.2)	23 (42.6)	29 (35.8)	18 (40.0)	12 (28.6)	30 (34.5)	14 (35.9)	19 (35.8)	33 (35.9)	13 (46.4)	16 (28.1)	29 (34.1)
Age, mean (SD)	30.7 (14.2)	30.0 (15.2)	30.3 (14.9)	30.1 (14.4)	29.1 (13.3)	30.1 (14.4)	30.4 (15.2)	29.8 (13.8)	30.1(14.3)	29.5 (16.4)	30.1(13.4)	29.9 (14.4)

Hyper—hyperdivergent; Normo—normodivergent; SN-MP—sella–nasion plane–mandibular plane; FH-MP—Frankfort horizontal plane–mandibular plane; PP-MP—palatal plane–mandibular plane; ODI—overbite depth indicator; SD—standard deviation.

**Table 2:** OHIP-14 according to hyperdivergent or normodivergent pattern defined by SN-MP, for overall participants (*n* = 81).

OHIP-14, Mean (SD)	SN-MP	SN-MP	*p*-Value *	Total (*n* = 81)	Adjusted Model for Sex and Age [SE] (*p*-Value)
	Hyperdivergent (*n* = 27)	Normodivergent (*n* = 54)			
Total	15 (11.0)	13.2 (11.3)	0.4046	13.8 (11.2)	**−0.15 [0.06] (0.010)**
Domains					
Functional limitation	0.7 (1.1)	0.5 (1.0)	0.5783	0.6 (1.0)	0.04 [0.21] (0.851)
Physical pain	1.6 (1.3)	1.4 (1.3)	0.8716	1.4 (1.3)	−0.01 [0.13] (0.966)
Psychological discomfort	1.9 (1.5)	1.7 (1.5)	0.2634	1.8 (1.5)	**−0.30 [0.12] (0.010)**
Physical disability	0.8 (1.1)	0.7 (1.1)	0.7395	0.8 (1.1)	0.02 [0.17] (0.930)
Psychological disability	1.5 (1.3)	1.3 (1.4)	0.7308	1.4 (1.4)	−0.24 [0.13] (0.079)
Social disability	0.5 (1.0)	0.5 (0.9)	0.2452	0.5 (1.0)	−0.31 [0.23] (0.165)
Handicap	0.6 (1.0)	0.6 (1.0)	0.5520	0.6 (1.0)	0.06 [0.20] (0.752)

OHIP-14—Oral Health Impact Profile 14; SD—standard deviation; SE—standard error; SN-MP—sella–nasion plane–mandibular plane; * Mann–Whitney test for continuous variables, *p* < 0.05 denoted in bold.

**Table 3:** OHIP-14 according to hyperdivergent or normodivergent pattern defined by FH-MP, for overall participants (*n* = 87).

OHIP-14, Mean (SD)	FH-MP	FH-MP	*p*-Value *	Total (*n* = 87)	Adjusted Model for Sex and Age [SE] (*p*-Value)
	Hyperdivergent (*n* = 45)	Normodivergent (*n* = 42)			
Total	14.8 (11.1)	13.0 (11.2)	0.4458	13.9 (11.2)	0.04 [0.06] (0.562)
Domains					
Functional limitation	0.6 (1.1)	0.6 (1.0)	0.9131	0.6 (1.1)	0.13 [0.22] (0.548)
Physical pain	1.5 (1.3)	1.5 (1.3)	0.3854	1.5 (1.3)	0.08 [0.14] (0.578)
Psychological discomfort	1.97 (1.5)	1.5 (1.4)	0.6862	1.7 (1.5)	−0.03 [0.13] (0.798)
Physical disability	0.8 (1.1)	0.8 (1.1)	0.3385	0.8 (1.1)	−0.02 [0.19] (0.933)
Psychological disability	1.5 (1.4)	1.2 (1.3)	0.8935	1.3 (1.4)	−0.07 [0.14] (0.600)
Social disability	0.5 (1.0)	0.4 (0.9)	0.4068	0.5 (1.0)	0.10 [0.24] (0.682)
Handicap	0.6 (1.0)	0.6 (1.0)	0.3949	0.6 (1.0)	0.07 [0.23] (0.762)

OHIP-14—Oral Health Impact Profile 14; SD—standard deviation; SE—standard error; FH-MP—Frankfort horizontal plane–mandibular plane; * Mann–Whitney test for continuous variables.

**Table 4:** OHIP-14 according to hyperdivergent or normodivergent pattern defined by PP-MP, for overall participants (*n* = 92).

OHIP-14, Mean (SD)	PP-MP	PP-MP	*p*-Value *	Total (*n* = 92)	Adjusted Model for Sex and Age, [SE] (*p*-Value)
	Hyperdivergent (*n* = 39)	Normodivergent (*n* = 53)			
Total	13.4 (11.0)	14.7 (11.4)	0.5472	14.1 (11.3)	0.10 [0.06] (0.092)
Domains					
Functional limitation	0.5 (1.0)	0.6 (1.1)	0.7363	0.6 (1.1)	0.16 [0.20] (0.414)
Physical pain	1.3 (1.2)	1.6 (1.3)	0.3716	1.5 (1.3)	0.22 [0.13] (0.079)
Psychological discomfort	1.7 (1.4)	1.8 (1.5)	0.2970	1.8 (1.5)	0.01 [0.11] (0.937)
Physical disability	0.8 (1.1)	0.8 (1.1)	0.2376	0.8 (1.1)	−0.02 [0.17] (0.898)
Psychological disability	1.3 (1.3)	1.4 (1.4)	0.8987	1.3 (1.4)	0.10 [0.13] (0.449)
Social disability	0.5 (1.0)	0.5 (1.0)	0.4350	0.5 (1.0)	0.06 [0.21] (0.788)
Handicap	0.6 (1.0)	0.7 (1.0)	0.5280	0.6 (1.0)	0.15 [0.19] (0.433)

OHIP-14—Oral Health Impact Profile 14; SD—standard deviation; SE—standard error; PP-MP—palatal plane–mandibular plane; * Mann–Whitney test for continuous variables.

**Table 5:** OHIP-14 according to hyperdivergent or normodivergent pattern defined by ODI, for overall participants (*n* = 85).

OHIP-14, Mean (SD)	ODI	ODI	*p*-Value *	Total (*n* = 85)	Adjusted Model for Sex and Age, [SE] (*p*-Value)
	Hyperdivergent (*n* = 28)	Normodivergent (*n* = 57)			
Total	13.1 (11.3)	14.6 (8.8)	0.7516	14.2 (11.1)	0.04 [0.06] (0.493)
Domains					
Functional limitation	0.5 (1.0)	0.6 (1.1)	0.6035	0.6 (1.1)	0.04 [0.22] (0.846)
Physical pain	1.2 (1.2)	1.6 (1.3)	0.4698	1.5 (1.3)	0.18 [0.14] (0.204)
Psychological discomfort	1.7 (1.4)	1.8 (1.5)	0.6392	1.8 (1.4)	−0.00 [0.12] (0.977)
Physical disability	0.7 (1.1)	0.8 (1.2)	0.7971	0.8 (1.1)	0.04 [0.19] (0.802)
Psychological disability	1.3 (1.3)	1.4 (1.4)	0.9279	1.4 (1.4)	0.07 [0.15] (0.628)
Social disability	0.6 (1.2)	0.4 (0.9)	0.4175	0.5 (1.0)	−0.36 [0.23] (0.128)
Handicap	0.6 (1.1)	0.7 (1.0)	0.7007	0.7 (1.0)	0.15 [0.21] (0.484)

OHIP-14—Oral Health Impact Profile 14; SD—standard deviation; SE—standard error; ODI—overbite depth indicator; * Mann–Whitney test for continuous variables.

## Data Availability

Data may be available upon reasonable request.

## Supplementary Materials

The following supporting information can be downloaded at: https://www.mdpi.com/article/10.3390/jcm13030665/s1, Table S1: STROBE Statement.; Table S2: Comparison of sagittal differences (through ANB angle) between vertical dimension categories.

## References

[1] Sun L., Wong H.M., McGrath C.P. (2017). Relationship between the severity of malocclusion and oral health related quality of life: A systematic review and meta-analysis. Oral Health Prev. Dent..

[2] Bock J.J., Odemar F., Fuhrmann R.A.W. (2009). Assessment of quality of life in patients undergoing orthognathic surgery. J. Orofac. Orthop..

[3] Göelzer J.G., Becker O.E., Junior O.L.H., Scolari N., Melo M.F.S., Heitz C., de Oliveira R.B. (2014). Assessing change in quality of life using the Oral Health Impact Profile (OHIP) in patients with different dentofacial deformities undergoing orthognathic surgery: A before and after comparison. Int. J. Oral Maxillofac. Surg..

[4] Rustemeyer J., Martin A., Gregersen J. (2012). Changes in quality of life and their relation to cephalometric changes in orthognathic surgery patients. Angle Orthod..

[5] Slade G.D., Spencer A.J. (1994). Development and evaluation of the Oral Health Impact Profile. Community Dent. Health.

[6] Slade G.D. (1997). Derivation and validation of a short-form oral health impact profile. Community Dent. Oral Epidemiol..

[7] Locker D., Allen F. (2007). What do measures of “oral health-related quality of life” measure?. Community Dent. Oral Epidemiol..

[8] Riva F., Seoane M., Reichenheim M.E., Tsakos G., Celeste R.K. (2022). Adult oral health-related quality of life instruments: A systematic review. Community Dent. Oral Epidemiol..

[9] Leite P.C.C., Camarini E.T., Filho L.I., Pavan Â.J., Farah G.J., da Silva M.B. (2004). Estudo epidemiológico das deformidades dentofaciais de Maringá/PR-1997/2003. Pesqui. Bras. Odontopediatria Clín. Integr..

[10] Ong M.A.H. (2004). Spectrum of dentofacial deformities: A retrospective survey. Ann. Acad. Med. Singap..

[11] Alanko O.M.E., Svedstrm-Oristo A.L., Tuomisto M.T. (2010). Patients’ perceptions of ortho gnathic treatment, well-being, and psychological or psychiatric status: A systematic review. Acta Odontol. Scand..

[12] Eslamipour F., Najimi A., Tadayonfard A., Azamian Z. (2017). Impact of Orthognathic Surgery on Quality of Life in Patients with Dentofacial Deformities. Int. J. Dent..

[13] Gomes A.M.P., Garbin C.A.S., Ferraz F.W.S., Saliba T.A., Garbin A.J.I. (2019). Dentofacial Deformities and Implications on Quality of Life: A Presurgical Multifactorial Analysis in Patients Seeking Orthognathic Surgical Treatment. J. Oral Maxillofac. Surg..

[14] Frejman M.W., Vargas I.A., Rösing C.K., Closs L.Q. (2013). Dentofacial deformities are associated with lower degrees of self-esteem and higher impact on oral health-related quality of life: Results from an observational study involving adults. J. Oral Maxillofac. Surg..

[15] Feragen K.B., Stock N.M. (2017). Psychological adjustment to craniofacial conditions (excluding oral clefts): A review of the literature. Psychol. Health.

[16] Alanko O.M.E., Svedström-Oristo A.L., Peltomäki T., Kauko T., Tuomisto M.T. (2014). Psychosocial well-being of prospective orthognathic-surgical patients. Acta Odontol. Scand..

[17] Rusanen J., Lahti S., Tolvanen M., Pirttiniemi P. (2010). Quality of life in patients with severe malocclusion before treatment. Eur. J. Orthod..

[18] Schendel S.A., Eisenfeld J., Bell W.H., Epker B.N., Mishelevich D.J. (1976). The long face syndrome: Vertical maxillary excess. Am. J. Orthod..

[19] Bell W.H., Creekmore T.D., Alexander R.G. (1977). Surgical correction of the long face syndrome. Am. J. Orthod..

[20] Ali U.S., Sukhia R.H., Fida M., Kamal A.T., Abbas A. (2022). Influence of incisor inclination and anterior vertical facial height on facial attractiveness in an adult Asian male. Am. J. Orthod. Dentofac. Orthop..

[21] Arqoub S.H.A., Al-Khateeb S.N. (2011). Perception of facial profile attractiveness of different antero-posterior and vertical proportions. Eur. J. Orthod..

[22] Morgan L.K., Kisley M.A. (2014). The effects of facial attractiveness and perceiver’s mate value on adaptive allocation of central processing resources. Evol. Hum. Behav..

[23] Lee S., McGrath C., Samman N. (2007). Quality of life in patients with dentofacial deformity: A comparison of measurement approaches. Int. J. Oral Maxillofac. Surg..

[24] Elm E.V., Altman D.G., Egger M., Pocock S.J., Gøtzsche P.C., Vandenbroucke J.P. (2007). The Strengthening the Reporting of Observational Studies in Epidemiology (STROBE) statement: Guidelines for reporting observational studies. PLoS Med..

[25] Pereira D., Machado V., Botelho J., Proença L., Rua J., Lemos C., Mendes J.J., Delgado A.S. (2021). Impact of malocclusion, tooth loss and oral hygiene habits on quality of life in orthodontic patients: A cross-sectional study. Int. J. Environ. Res. Public. Health.

[26] Andiappan M., Gao W., Bernabé E., Kandala N.B., Donaldson A.N. (2015). Malocclusion, orthodontic treatment, and the Oral Health Impact Profile (OHIP-14): Systematic review and meta-analysis. Angle Orthod..

[27] Afonso A., Silva R.M.I., Frias-Bulhosa J. (2017). Qualidade de Vida Relacionada Com a Saude Oral: Validaçao Portuguesa de OHIP-14. Soc. Port. Psicol. Saúde.

[28] Riedel R. (1952). The relation of maxillary structures to cranium in malocclusion and in normal occlusion. Angle Orthod..

[29] Sato S. (1987). Alterations of Occlusal Plane due to Posterior Discrepancy related to development of malocclusion—Introduction to denture frame analysis. Bull. Kanagawa Dent. Col..

[30] Bjork A., Lundstrom A. (1960). The Relationship of the Jaws to the Cranium.

[31] Kim Y.H. (1974). Overbite depth indicator with particular reference to anterior open-bite. Am. J. Orthod..

[32] Antoun J.S., Thomson W.M., Merriman T.R., Rongo R., Farella M. (2017). Impact of skeletal divergence on oral health-related quality of life and self-reported jaw function. Korean J. Orthod..

[33] Meger M.N., Fatturi A.L., Gerber J.T., Weiss S.G., Rocha J.S., Scariot R., Wambier L.M. (2021). Impact of orthognathic surgery on quality of life of patients with dentofacial deformity: A systematic review and meta-analysis. Br. J. Oral Maxillofac. Surg..

[34] de Araujo C.M., Schroder A.G.D., de Araujo B.M.M., Calvacante-Leão B.L., Stechman-Neto J., Zeigelboim B.S., Santos R.S., Guariza-Filho O. (2020). Impact of orthodontic-surgical treatment on quality of life: A meta-analysis. Eur. J. Orthod..

[35] Tuk J.G., Lindeboom J.A., Tan M.L., de Lange J. (2022). Impact of orthognathic surgery on quality of life in patients with different dentofacial deformities: Longitudinal study of the Oral Health Impact Profile (OHIP-14) with at least 1 year of follow-up. Oral Maxillofac. Surg..

[36] Ryan F.S., Barnard M., Cunningham S.J. (2012). Impact of dentofacial deformity and motivation for treatment: A qualitative study. Am. J. Orthod. Dentofac. Orthop..

[37] de Oliveira E.G.S., Pinzan-Vercelino C.R.M. (2013). Comparative evaluation of cephalometric and occlusal characteristics between the Long Face pattern and Pattern I. Dent. Press J. Orthod..

[38] Curto A., Albaladejo A., Alvarado-Lorenzo A. (2022). Oral-Health-Related Quality of Life (OHRQoL) and Anterior Open Bite in Adult Patients: A Case-Control Study. Healthcare.

[39] Plaza S.P., Reimpell A., Silva J., Montoya D. (2019). Relationship between skeletal class II and class III malocclusions with vertical skeletal pattern. Dent. Press J. Orthod..

[40] Cochrane S.M., Cunningham S.J., Hunt N.P. (1999). A comparison of the perception of facial profile by the general public and 3 groups of clinicians. Int. J. Adult Orthodon. Orthognath. Surg..

[41] Johnston D.J., Hunt O., Johnston C.D., Burden D.J., Stevenson M., Hepper P. (2005). The influence of lower face vertical proportion on facial attractiveness. Eur. J. Orthod..

[42] Pabari S., Moles D.R., Cunningham S.J. (2011). Assessment of motivation and psychological characteristics of adult orthodontic patients. Am. J. Orthod. Dentofac. Orthop..

[43] Goulart M.S., Filho L.C., Conti A.C.C.F., Pedrin R.R.A., Ladewig V.M., Cardoso M.A. (2019). Evaluation of facial esthetics in long-faced white Brazilian middle school students. Am. J. Orthod. Dentofac. Orthop..

[44] Ahmed M., Shaikh A., Fida M. (2016). Diagnostic performance of various cephalometric parameters for the assessment of vertical growth pattern. Dent. Press J. Orthod..

[45] Esperão P.T.G., de Oliveira B.H., Almeida M.A.O., Kiyak H.A., Miguele J.A.M. (2010). Oral health-related quality of life in orthognathic surgery patients. Am. J. Orthod. Dentofac. Orthop..

[46] Cohen L.K., Jago J.D. (1976). Toward the formulation of sociodental indicators. Int. J. Health Serv..

