# Supplementary material for: Vertical Dentofacial Skeletal Divergency Is Not Linked with Oral Health-Related Quality of Life
Source: J Clin Med. 2024 Jan 24;13(3):665. doi: 10.3390/jcm13030665 (PMC10856730; doi:10.3390/jcm13030665)
Supplement: Supplementary file 1 [file jcm-13-00665-s001.zip › Table S2.pdf]

Table S2. Comparison of sagittal differences (through ANB angle) between vertical dimension categories.

Variable	SN - MP		p-value	FH - MP		p-value	PP - MP		p-value	ODI		p-value
	**Hyper (n = 27)**	**Normo (n = 54)**		**Hyper (n = 45)**	**Normo (n = 42)**		**Hyper (n = 39)**	**Normo (n = 53)**		**Hyper (n = 28)**	**Normo (n = 57)**	
ANB	4.3 (2.7)	3.2 (2.4)	0.519	4.6 (2.63)	3.51 (2.49)	0.572	4.2 (2.8)	3.6 (2.3)	0.317	2.0 (2.8)	4.2 (2.3)	0.143

SN-MP - Sella-Nasion plane - Mandibular Plane; FH-MP - Frankfort Horizontal Plane - Mandibular Plane; PP-MP – Palatal Plane - Mandibular Plane; ODI - Overbite Depth Indicator; Hyper - Hyperdivergent; Normo - Normodivergent; SD - Standard Deviation.

* Mann-Whitney for continuous variables, chi-square test for categorical variables, $p < 0.05$ denoted in bold.
